# Outcomes of 38 patients with PFIC3: Impact of genotype and of response to ursodeoxycholic acid therapy

**DOI:** 10.1016/j.jhepr.2023.100844

**Published:** 2023-07-13

**Authors:** Emmanuel Gonzales, Antoine Gardin, Marion Almes, Amaria Darmellah-Remil, Hanh Seguin, Charlotte Mussini, Stéphanie Franchi-Abella, Mathieu Duché, Oanez Ackermann, Alice Thébaut, Dalila Habes, Bogdan Hermeziu, Martine Lapalus, Thomas Falguières, Jean-Philippe Combal, Bernard Benichou, Sonia Valero, Anne Davit-Spraul, Emmanuel Jacquemin

**Affiliations:** 1 Pediatric Hepatology and Liver Transplantation, National Reference Centre for Biliary Atresia and Genetic Cholestasis, FILFOIE, ERN RARE LIVER, France; 2 Pathology, Bicêtre Hospital, Assistance Publique – Hôpitaux de Paris, University Paris-Saclay, Le Kremlin-Bicêtre, France; 3 Pediatric Radiology, Bicêtre Hospital, Assistance Publique – Hôpitaux de Paris, University Paris-Saclay, Le Kremlin-Bicêtre, France; 4 Vivet Therapeutics, Paris, France; 5 Biochemistry; Bicêtre Hospital, Assistance Publique – Hôpitaux de Paris, University Paris-Saclay, Le Kremlin-Bicêtre, France; 6 Inserm U1193, Hepatinov, University Paris-Saclay, Orsay, France

**Keywords:** *ABCB4*, MDR3, intrahepatic cholestasis of pregnancy, biliary lithiasis, liver transplantation, hepatocellular carcinoma

## Abstract

**Background & Aims:**

Progressive familial intrahepatic cholestasis type 3 (PFIC3) is a rare liver disease caused by biallelic variations in *ABCB4*. Data reporting on the impact of genotype and of response to ursodeoxycholic acid (UDCA) therapy on long-term outcomes are scarce.

**Methods:**

We retrospectively describe a cohort of 38 patients with PFIC3 with a median age at last follow-up of 19.5 years (range 3.8–53.8).

**Results:**

Twenty patients presented with symptoms before 1 year of age. Thirty-one patients received ursodeoxycholic acid (UDCA) therapy resulting in serum liver test improvement in 20. Twenty-seven patients had cirrhosis at a median age of 8.1 years of whom 18 received a liver transplant at a median age of 8.5 years. Patients carrying at least one missense variation were more likely to present with positive (normal or decreased) canalicular MDR3 expression in the native liver and had prolonged native liver survival (NLS; median 12.4 years [range 3.8-53.8]). In contrast, in patients with severe genotypes (no missense variation), there was no detectable canalicular MDR3 expression, symptom onset and cirrhosis occurred earlier, and all underwent liver transplantation (at a median age of 6.7 years [range 2.3–10.3]). The latter group was refractory to UDCA treatment, whereas 87% of patients with at least one missense variation displayed an improvement in liver biochemistry in response to UDCA. Biliary phospholipid levels over 6.9% of total biliary lipid levels predicted response to UDCA. Response to UDCA predicted NLS.

**Conclusions:**

Patients carrying at least one missense variation, with positive canalicular expression of MDR3 and a biliary phospholipid level over 6.9% of total biliary lipid levels were more likely to respond to UDCA and to exhibit prolonged NLS.

**Impact and implications:**

In this study, data show that genotype and response to ursodeoxycholic acid therapy predicted native liver survival in patients with PFIC3 (progressive familial intrahepatic cholestasis type 3). Patients carrying at least one missense variation, with positive (decreased or normal) immuno-staining for canalicular MDR3, and a biliary phospholipid level over 6.9% of total biliary lipids were more likely to respond to ursodeoxycholic acid therapy and to exhibit prolonged native liver survival.

## Highlights

• 71% of patients had cirrhosis at a median age of 8.1 years of whom 66% received a liver transplant at a median age of 8.5 years.
• Patients with a severe genotype (no missense variation) did not respond to UDCA treatment.
• 87% of patients with at least one missense variation responded partially or fully to UDCA treatment.
• Biliary phospholipid level over 6.9% of total biliary lipids predicted response to UDCA therapy.
• Severity of genotypes and response to UDCA treatment predicted native liver survival.

## Graphical abstract

**Figure:**
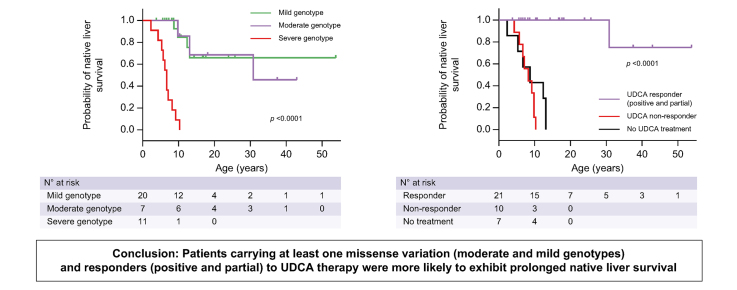

## Introduction

Diseases associated with MDR3/ABCB4 deficiency were identified more than 20 years ago, both in adult and pediatric patients.^1^^,^^2^ These diseases are due to variations in the canalicular phospholipid transporter gene, namely ATP-binding cassette subfamily B member 4 (*ABCB4*). *ABCB4* encodes the multidrug resistance protein 3 (MDR3) which is expressed at the canalicular membrane of the hepatocyte where it acts as a phosphatidylcholine floppase. MDR3 is essential for biliary secretion of phospholipids (PL).^3^ Progressive familial intrahepatic cholestasis type 3 (PFIC3, OMIM#602347) which results from biallelic pathogenic variations in *ABCB4* constitutes the most severe phenotype related to MDR3 deficiency. Other phenotypes related to MDR3 deficiency classically include oral contraceptive induced cholestasis (OCIC), intrahepatic cholestasis of pregnancy (ICP3, OMIM#614972) and low phospholipid-associated cholelithiasis (LPAC) syndrome (also reported as gallbladder disease 1, OMIM#600803) and are due to monoallelic variation in *ABCB4* in most cases.[1], [2], [3] Monoallelic pathogenic variation in *ABCB4* can also lead to progressive liver disease during adulthood.[4], [5], [6] Lastly, MDR3 deficiency is associated with an increased risk of developing hepatobiliary cancer, especially cholangiocarcinoma during adulthood.^6^ The pathophysiology of MDR3 deficiency is thought to result from (i) a lack of PL protection in the bile against the detergent effect of bile salts, with ensuing damage to hepatocytes and biliary epithelium, bile ductular proliferation, and progressive portal fibrosis; (ii) an increased biliary lithogenicity leading to a cholesterol gallstone disease which may result in bile duct obstruction.^3^

Patients with PFIC3 usually exhibit jaundice, hepatosplenomegaly, discolored stools, and pruritus during childhood. Most patients progress to cirrhosis, portal hypertension (PHT), and liver failure during the first two decades of life.^1^^,^^3^^,^[7], [8], [9], [10], [11], [12] Oral administration of ursodeoxycholic acid (UDCA), the first-line treatment for PFIC3, has been reported to improve clinical symptoms and serum liver tests in some affected children but the impact of this treatment, as well as of genotype severity, on long-term outcomes, including native liver survival (NLS), is not known.^1^^,^^3^^,^^8^^,^[10], [11], [12] A significant proportion of patients require liver transplantation (LT), the only curative treatment for PFIC3.^1^^,^^3^^,^[10], [11], [12] So far, only a limited number of incompletely characterized PFIC3 case series with relatively short follow-up time have been described in the literature. Therefore, an in-depth characterization of a cohort of patients with PFIC3 is lacking. In this study, we report on the outcomes of 38 well-characterized patients, focusing on response to UDCA therapy, genotype-phenotype correlation and NLS.

## Patients and methods

### Patients

We retrospectively reviewed medical records of all patients with PFIC3 referred to our tertiary care reference center between 1986 and 2021. Only patients in whom two disease-causing variations in the *ABCB4* gene were identified were included in the study. Patient data was collected from disease presentation until the last follow-up visit with native liver. In liver transplanted patients, data on pregnancy, retransplantation, and death were collected. In some patients in whom a post-LT serum sample was available, screening for anti-MDR3 post-LT alloimmunization was performed using a previously described procedure.^13^ Collected data included molecular analyses of *ABCB4* results, clinical findings including pruritus (graded as present or absent), serum liver tests, serum bile acids (BA), imaging, upper gastrointestinal endoscopy (UGIE) findings, liver histological studies including MDR3 immunostaining and biliary lipid composition analyses.^1^ Imaging studies included abdominal ultrasonography, percutaneous transhepatic cholecystography, endoscopic retrograde cholangiography and magnetic resonance cholangiography. The following criteria were systematically reviewed by study investigators (EJ, EG) to determine if and when the patient developed a cirrhotic status: competing events, including liver histology (F4), ultrasound and UGIE findings (signs of PHT) and hypersplenism. In some patients, liver and spleen stiffness were measured using liver elastography supersonic shear imaging technology.^14^

### Liver histology and MDR3 immunostaining studies

All available liver histological analysis reports were collected and used to calculate retrospectively a fibrosis score and an activity score according to the Metavir scoring system. In addition, the presence of ductular proliferation, ductular plug, giant cell transformation and of hepatocellular and canalicular cholestasis was assessed. All liver samples were analyzed in the same pathology laboratory. MDR3 immunostaining was performed as previously reported, either as part of the initial analysis of the liver sample specimen or retrospectively for the purpose of the present study; the canalicular staining intensity of MDR3 was evaluated and graded as normal, decreased, or absent.^1^

### Molecular study of *ABCB4* gene and classification of genotypes

From 1998 to 2015, the *ABCB4* gene was sequenced by the Sanger technique.^1^ After 2015, *ABCB4* was analyzed using a next-generation sequencing technique.^15^ All variations were classified according to the American College of Medical Genetics criteria.^15^ To study genotype-phenotype correlation, patient genotypes were classified as severe, moderate or mild, based on the presence of zero, one or two missense variations, respectively. Missense variations altering the start codon or modifying splicing were assimilated to a severe variation. Small in-frame deletions were grouped together with missense variations.

### UDCA treatment and assessment of response to UDCA

From 1988, patients were treated with oral UDCA at the daily dose of 600 mg/m^2^ of body area (*e.g.* the dose in mg/kg ranged from 28 mg/kg in a 10 kg child to 15 mg/kg in a 70 kg adult patient).^1^ In some patients, UDCA therapy was transiently interrupted to assess treatment efficacy. A positive response to UDCA treatment was defined as normalization of serum alanine aminotransferase and gamma-glutamyltransferase activities, and of total and conjugated bilirubin values when available, within 6 to 12 months after starting UDCA. A partial response was defined as reduction in liver enzymes without reaching normal values. No improvement of these biological parameters constituted a negative response.^1^

### Ethical approval

The study was conducted in accordance with guidelines of the Declaration of Helsinki and in compliance with French regulatory authorities for data handling and processing (CNIL registration number: 2217140 v 0). The study protocol was approved by the independent ethics committee of the French-speaking Group for Pediatric Hepatology Gastroenterology and Nutrition.

### Statistical analyses

Results are reported as median (range). Survival curves were calculated according to the Kaplan-Meier method and comparisons between groups of patients were made using a log-rank test. Confidence intervals were two-sided, with a significance level set at α <0.05. For NLS, relevant events were liver transplantation or death without transplantation. For cirrhosis-free survival, the relevant event was cirrhosis. Receiver-operating characteristic (ROC) analysis was performed to assess the relationship between biliary phospholipid (PL)/total biliary lipid (TBL) ratio values and response to UDCA treatment (area under the ROC curve significance; null hypothesis: AUC = 0.5). All statistical analyses were conducted using GraphPad Prism 9.5.0 (Dotmatics, Boston, USA).

## Results

### Patients and relatives

We included 38 patients, including 21 males, from 33 families, with a diagnosis of PFIC3 (Table S1). Among the 33 families, consanguinity was present in 14, absent in 14 and unknown in five; nearly half of the patients (n = 18) were born to consanguineous parents. Partial data concerning 14 patients (patients 4-7, 12-16, 21, 22, 25, 8, 18) were previously reported.^1^^,^^15^^,^^16^ Medical events compatible with MDR3 deficiency-related liver disorders were recorded in relatives belonging to 20 out of the 33 families. These medical events include PFIC3 phenotype (n = 3, one brother of patient 18 and two cousins of patient 4), episode of ICP (seven mothers [families B, D, F, G, W, X, AF], one grandmother [family F] and one aunt [family AE]), cholelithiasis including LPAC syndrome (five parents [families J, U, X, Y, AF], one sister [family P] and 13 additional relatives from 11 families [families F, G, J, L, U, X, Y, AA, AE, AF, AG]), cholangiocarcinoma (n = 2, one father [family C] and one grandfather [family AG]) and hepatocellular carcinoma (n = 1, one uncle [family G]). In addition, cirrhosis, cause unknown, was reported in one grandparent (family AB) and two aunts or uncles (family E and Q).

### Molecular studies of *ABCB4* and classification of genotypes

*ABCB4* genotyping identified 41 different variations (Table S1). Twenty-two (58%) patients were homozygous including the 18 patients born to consanguineous parents and 16 (42%) had a compound heterozygous status. Eleven patients had a genotype classified as severe (patients 1-11). Seven patients had a moderate genotype (patients 12-18). Patient 17 carried a missense variation altering the start codon which was assimilated to a severe variation and the patient genotype was classified as moderate. The remaining 20 patients had a mild genotype (patients 19-38) as these patients carried two missense variations. In total, 27 patients carried at least one missense variation (patients with moderate and mild genotypes). All variations were classified as pathogenic (class 5) or likely pathogenic (class 4) (Table S1).

### Symptom onset and evaluation at referral

The clinical presentation of the patients is summarized in Table 1 and Table S2. Median age at symptom onset was 0.9 years (range 0.1–23.3). Five patients (13%) were older than 10 years, including one adult (>18 years) patient, at the time of symptom onset. Age at referral ranged from 0.1 to 26.3 years (median 3.3 years). At that time, hepatomegaly (n = 31, 82%), pruritus (n = 30, 79%), and splenomegaly (n = 26, 68%) were the most frequent symptoms recorded. Jaundice was present in 11 patients (29%); a history of transient neonatal jaundice was recorded in four additional patients. Stool discoloration was recorded in six patients, including four with jaundice. Three patients, referred after the age of 15 years, initially presented with gallstone disease, including two female patients, one who also presented with ICP and one with OCIC. Biological data at the time of referral is detailed in Table S3. At referral, all patients had abnormal serum liver tests including elevated alanine aminotransferase (median; range) (180; 44–957; normal <50 IU/L) and gamma-glutamyltransferase (230; 75–958; normal <40 IU/L). Serum total bilirubin (22; 4–570; normal <17 μmol/L) or conjugated bilirubin (20; 1–245; normal <5 μmol/L) levels were increased in 28 patients. Serum BA levels measured in 14 out of the 38 patients were increased in all (92; 15–304; normal <10 μmol/L). At least one liver specimen collected before or shortly after the time of referral was available in 29 patients (Table S4). Histological reports were consistent with cirrhosis in 10 patients. Significant fibrosis (>F2) was recorded in 10 additional patients, including one with F3/F4 fibrosis and PHT who was considered to have cirrhosis (patient 26). Ductular proliferation was observed in 25 out of the 29 patients. Inflammation (>A1) and cholestasis (hepatocellular or canalicular) were observed in 12 and 10 patients, respectively (Table S4). MDR3 immunostaining analysis performed in the native livers of 23 patients showed a negative canalicular staining in nine patients (39%), a decreased staining in nine (39%) and a normal staining in five (22%) (Table 1 and Table S2).

**Table 1:** **Overview of canalicular staining of MDR3, biliary phospholipid analysis, clinical symptoms, UDCA treatment and treatment response, cirrhotic status and outcomes in 38 patients with PFIC3, according to genotype classification**.

Patient	Canalicular MDR3 staining	Biliary PL (% of TBL) (N 19-24)	Age at first symptoms/at referral (yr)	Symptoms at first evaluation in referral center	Age at UDCA onset (yr) and response to UDCA	Age at cirrhosis (yr)	Age at first LT (yr)	Indication for LT	Age at last f/u or death (yr)	Status at last f/u
**Severe genotype**										

Patients 1-11 (n = 11)	NA = 5Negative = 6	NA = 83.8 (2.7-4.5)	0.7 (0.1-5.7) 2.8 (0.1-5.9)	J = 5, TJ = 4, H = 10, S = 9, SD = 3, As = 2, P = 8	3.9 (0.3-7.1) NA = 3, Negative = 8	Cirrhosis = 11 (F4 = 6, PHT = 11) 5.7 (2.3-8.1)	LT = 11 6.7 (2.3-10.3)	Cirrhosis = 11 P = 2, HPS = 1 Re-LT = 4	28 (7.8-39.6)	Dead = 2 Alive = 9

**Moderate genotype**										

Patients 12-18 (n = 7)	NA = 3 Decreased = 3 Absent = 1	NA = 2 7.3 (4.7-14.5)	3.9 (0.7-17.6) 12.6 (0.7-17.9)	J = 3, H = 5, S = 5, SD = 1, As = 1, P = 6, Bl = 2, EHL = 2, CHOL = 1, SPSD = 1, OCIC = 1	12.2 (4.2-17.9) NA = 1, Positive = 3, Partial = 2, Negative = 1	Cirrhosis = 6 (F4 = 5, PHT = 5) 8.1 (3.9-17.9)	LT = 3 13.1 (9.8-30.8)	Cirrhosis = 3 HCC = 1 Re-LT = 1	31.4 (10.3-42.9)	Dead = 0 Alive = 7

**Mild genotype**										

Patients 19-38 (n = 20)	NA = 7 Normal = 5 Decreased = 6 Absent = 2	NA = 15 8.6 (4.4-13.2)	1.1 (0.1-23.3) 3 (1-26.3)	J = 3, TJ = 1, H = 16, S = 12, SD = 2, As = 2, P = 16, Bl = 1, EHL = 3, CHOL = 1, ICP = 1	2.6 (1.2-27.3) NA = 3, Positive = 1, Partial = 3, Negative = 1	Cirrhosis = 10 (F4 = 4, PHT = 9) 8.5 (1.6-51.4)	LT = 4 11.1 (8.7-13.1)	Cirrhosis = 4 Re-LT = 1	13.5 (3.8-53.8)	Dead = 1 Alive = 19

As, ascites; Bl, gastrointestinal bleeding due to portal hypertension; CHOL, cholecystectomy; EHL, extrahepatic biliary lithiasis; F4, cirrhosis at the pathological examination of liver sample allowing to establish the status of cirrhosis; f/u, follow-up; H, hepatomegaly; HCC, hepatocellular carcinoma; HPS, hepatopulmonary syndrome; ICP, intrahepatic cholestasis of pregnancy; J, jaundice; LT, liver transplantation; NA, not available or not applicable; OCIC, oral contraceptive induced cholestasis; P, pruritus; PL, phospholipids; PHT, portal hypertension; Re-LT, retransplantation; S, splenomegaly; SD, stool discoloration; SPSD, surgical portosystemic derivation; TBL, total biliary lipids; TJ, transient jaundice; UDCA, ursodeoxycholic acid.

At least one cholangiogram was obtained in 24 patients after a percutaneous transhepatic cholecystography (n = 18), an endoscopic retrograde cholangiopancreatography (n = 3) or a magnetic resonance cholangiopancreatography (n = 3). The cholangiogram was normal in 18 patients. In three patients (patients 8, 18, 33), including two previously reported patients, the cholangiogram was consistent with secondary sclerosing cholangitis.^15^ In four other patients, the cholangiogram showed an enlarged gallbladder. Liver ultrasonography showed an enlarged gallbladder in one additional patient. Biliary lipid analysis was performed in 13 patients in whom bile has been sampled (Table S5). PL concentrations were low in all patients with a median value of 2.2 mmol/L (range 0.1–14.4), resulting in a decreased PL/TBL ratio (median 7.3%; range 2.7%–14.5%) and increased BA/PL and cholesterol/PL ratios.

### Patient evolution and outcome

**Fig. 1:**
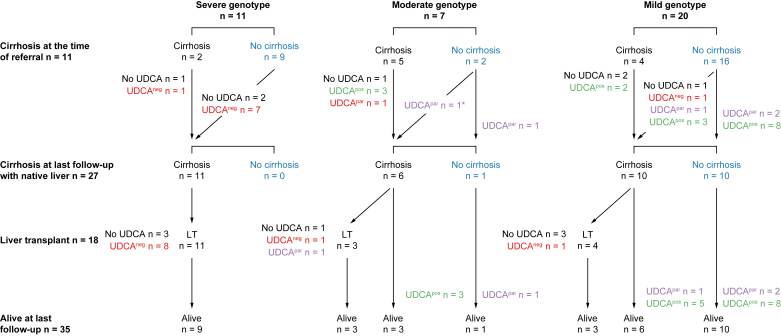
Flowchart of the outcome of the 38 patients with PFIC3 according to genotype classification and biochemical response to UDCA. LT, liver transplantation; UDCA, ursodeoxycholic acid; UDCA^neg^, negative response to UDCA (red color); UDCA^par^, partial response to UDCA (purple color); UDCA^pos^, positive response to UDCA (green color). ∗This patient had cirrhosis before being started with UDCA therapy. Absence of cirrhosis is indicated in blue color.

The median age at last follow-up or death was 19.5 years (range 3.8–53.8) and the median duration of follow-up was 14.6 years (range 1.7–33.7). Evolution, including response to UDCA, is summarized in Fig. 1 and Table 1, and detailed in Table S2. Biological data at last follow-up with native liver is provided in Table S3. The results of histological studies obtained during follow-up, including native liver collected at the time of LT are provided in Table S4.

UDCA therapy was initiated in 31 patients (82%), including seven who presented with cirrhosis at the time of referral, at a median age of 4.2 years (range 0.3–27.3). Sixteen patients (52%) showed a positive response to UDCA therapy; partial response was seen in five patients (16%) and negative response in 10 (32%) (Fig. 1, Table 1, Table 2, and Table S2). Four patients, three with a positive response and one with a partial response, were challenged with treatment discontinuation that resulted in worsening of serum liver tests which improved again after restarting UDCA (Table S2). Seven additional patients experienced episodes of poor compliance during which a worsening of serum liver tests was observed in three patients with a positive response and in two with a partial response to UDCA treatment. A normalization or improvement of liver function tests was observed in these patients when compliance to treatment improved. The two remaining patients had a negative response to UDCA treatment. Pruritus was reported in 32 (84%) patients over the disease course. Medical treatments were given to 31 patients (Table 1, Table S2). Among them, UDCA was started as a monotherapy in 23 patients and resulted in complete resolution of pruritus in 17 patients, partial resolution in four and no improvement in two.

**Table 2:** **Serum liver tests in patients with PFIC3 according to the response to UDCA therapy before initiation of UDCA and 6 to 12 months after initiation of UDCA**.

	GGT (normal <40 IU/L)		ALT (normal <50 IU/L)		Total bilirubin (normal <17 μmol/L)		Conjugated bilirubin (normal <5 μmol/L)	
Response to UDCA	at UDCA	6-12 UDCA	at UDCA	6-12 UDCA	at UDCA	6-12 UDCA	at UDCA	6-12 UDCA
Positive, n = 16								
Median	193	15	207	26	11	7	2	2
Min	83	7	53	12	3	3	1	1
Max	1,321	37	729	50	112	17	69	5
Partial, n = 5								
Median	259	77	259	43	21	9	15	4
Min	81	15	55	16	6	5	3	1
Max	680	82	957	78	40	33	26	19
Negative, n = 10								
Median	483	390	292	137	33	53	21	34
Min	141	153	76	54	10	25	5	13
Max	958	1,737	460	515	336	570	250	144

ALT, alanine aminotransferase; GGT, gamma-glutamyltransferase; UDCA, ursodeoxycholic acid.

Based on findings at referral, 11 patients (29%) were considered to have cirrhosis (Table 1, Table S2 and Fig. 1). During follow-up, 16 additional patients reached a cirrhotic status. Overall, 27 of the 38 patients (71%) reached a cirrhotic status at a median age of 8.1 years (range 1.6–51.4). This cirrhotic status was supported by histological study of the liver (n = 2), by the presence of signs of PHT (n = 11) or by both criteria (n = 13). Cirrhosis was determined by imaging studies in one patient.

### Patients with cirrhosis who received a LT

LT from deceased donors was performed in 18 patients with cirrhosis (patients 1-11, 13, 15, 16, 20-22, 30) (47%) at a median age of 8.5 years (range 2.3–30.8). Seven patients did not receive UDCA, 10 had a negative response and one a partial response to UDCA (Fig. 1, Table 1, Table S2). Indications for LT were decompensated cirrhosis (n = 15), refractory pruritus (n = 2), and hepatocellular carcinoma (n = 1, patient 15). Among these 18 patients, prolonged jaundice and ascites were recorded in 17 and 12 patients, respectively. Liver failure was recorded in 11 patients, two of whom developed hepatic encephalopathy. Among these 18 patients, only one (patient 30) did not present with PHT. Two patients presented with gastrointestinal hemorrhage due to grade 3 varices and underwent a surgical portosystemic derivation. Endoscopic primary prophylaxis of variceal bleeding was successfully performed in three patients. UGIE performed in 13 additional patients showed grade 2 varices in two, grade 1 varices in eight and no varices in three patients. Extrahepatic gallstone disease was recorded in three patients before LT; in two additional patients, previously unidentified intrahepatic biliary lithiases were observed in native livers. Six patients underwent a second LT (Table 1, Table S2). Three of the 18 patients died 15 to 33 years after their first LT at the ages of 24.1, 39.5 and 39.6 years. The remaining patients were alive at last follow-up with a median age of 29.1 years (range 7.8–39.7). One transplanted female patient experienced one pregnancy and gave birth to a healthy child. Immunofluorescence staining of normal human liver sections with patient sera (patients 1-8, 10, 13, 16), collected after a median period of 10 years after LT (range 1.5-16), and using an anti-human IgG antibody to detect serum antibodies, showed no reactivity to a canalicular epitope.

### Patients with cirrhosis who did not receive a LT

The remaining nine patients (patients 12, 14, 18, 19, 25, 26, 32, 33, 35 – see Fig. 1, Table 1 and Table S2) with a cirrhotic status were alive with their native liver at a median age of 16.9 years (range 6.7–53.8). Among them, four patients presented with cirrhosis before UDCA treatment initiation. All nine of these patients had a positive response to UDCA except for patient 19, who showed a partial response and was not fully compliant to UDCA. Five patients exhibited significant PHT as indicated by persistent splenomegaly and hypersplenism. One patient (patient 12), presenting at referral with variceal hemorrhage due to grade 2 varices and ascites, was successfully managed by endoscopic and medical treatments. Endoscopic primary prophylaxis of variceal bleeding was successfully performed in three patients (patients 18, 33, 25) with grade 3, grade 2 and grade 1 varices, respectively. Over time, clinical signs of PHT regressed in three patients (patients 25, 26, 35), including one patient in whom results of histological study were consistent with a decrease in fibrosis from F3/4 to F2/3. Liver elastometry measurement values, available in three of these nine patients, ranged between 6.9 to 13.4 kPa (Table S2) and tended to decrease over time (data not shown). Spleen elastometry measurement values, available in these three patients, ranged from 21.5 to 41 kPa. In two female patients, PFIC3 was diagnosed around the age of 20 years in the setting of gallstone disease and OCIC (patient 14) or ICP (patient 19). These two patients underwent uneventful cholecystectomy; one of them also presented intrahepatic stones and later underwent multiple endoscopic retrograde procedures. Patient 14 gave birth to three healthy children after three pregnancies complicated with ICP. During the first pregnancy, UDCA therapy was interrupted leading to refractory pruritus requiring albumin dialysis.^16^ The two following ICP episodes occurred despite continuous UDCA therapy but were mild. Patient 19 gave birth to five healthy children after seven pregnancies, six of which were complicated with ICP but adherence to UDCA treatment was poor. At last follow-up, none of these nine patients was jaundiced, presented with decompensated cirrhosis, or was enlisted for LT.

### Patients without cirrhosis

Eleven patients (patients 17, 23, 24, 27-29, 31, 34, 36-38) were not considered to have cirrhosis (Fig. 1, Table 1 and Table S2). All were alive with their native liver at a median age of 10.6 years (range 3.8–25.7). All were treated with UDCA with a positive response in eight and a partial response in three. Among them, four patients (patients 28, 29, 34, 37) presented with splenomegaly which regressed over time. UGIE performed in four patients did not show varices. Liver elastometry measured in eight patients showed normal or slightly elevated values ranging between 5 and 8.7 kPa. One patient (patient 17) was diagnosed after he underwent a cholecystectomy at age 15.7 years. Five additional patients (patients 23, 24, 27, 31, 34) presented with gallstone disease, which proved transient in three.

### Outcome according to genotype

**Fig. 2:**
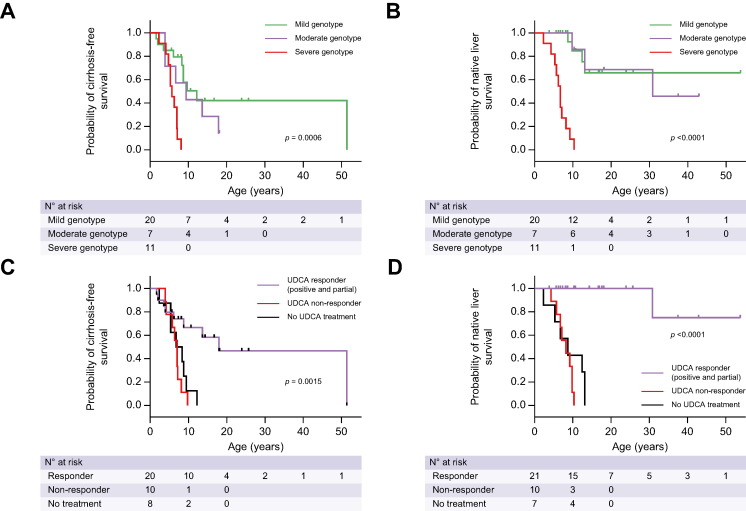
Cirrhosis-free survival and native liver survival according to genotypes and to UDCA therapy and response to this therapy. (A,B) Cirrhosis-free survival (A) (*p* = 0.0006; log-rank test) and native liver survival (B) (*p* <0.0001; log-rank test) per genotypic severity; (C,D) cirrhosis-free survival (C) (*p* = 0.0015; log-rank test) and native liver survival (D) (*p* <0.0001; log-rank test) per response to UDCA treatment and response to this treatment. UDCA, ursodeoxycholic acid.

First symptoms occurred at a median age of 0.7 (range 0.1-5.7), 3.9 (range 0.7-17.6), and 1.1 (range 0.1-23.3) years in patients with severe, moderate, and mild genotypes, respectively (Table 1). All patients (11/11) with severe genotypes showed evidence of cirrhosis at a median age of 5.7 years (range 2.3-8.1) as compared to 86% (6/7) of patients with moderate genotypes and 50% (10/20) of patients with mild genotypes who presented with cirrhosis at a median age of 8.1 years (range 3.9-17.9) and 8.5 years (range 1.6-51.4), respectively (Fig. 2A; *p* = 0.0006; Fig. S1A). All patients (11/11) with severe genotypes were transplanted at a median age of 6.7 years (range 2.3-10.3) compared to 43% (3/7) of patients with moderate genotypes and to 20% (4/20) of patients with mild genotypes who were transplanted at a median age of 13.1 (range 9.8-30.8) and 11.1 (range 8.7-13.1) years, respectively (Fig. 2B; *p* <0.0001; Fig. S1B). Patients carrying at least one missense variation (moderate and mild genotypes) had a median NLS of 12.4 years (range 3.8–53.8). All patients (6/6) with severe genotypes had negative canalicular MDR3 staining compared to 25% (1/4) and 15% (2/13) of patients with moderate and mild genotypes, respectively (Table 1 and Table S2). Normal canalicular MDR3 staining was only observed in patients with mild genotypes (5/13). Patients with severe genotypes presented with lower biliary PL/TBL ratios, with a median value of 3.8% (range 2.7-4.5) compared to patients with moderate (7.3%, range 4.7-14.5) and mild (8.6%, range 6.3-12.2) genotypes (Table S5). Response to UDCA therapy was negative in all patients (8/8) with severe genotypes (Table 1 and Table S2). By contrast, 83% (5/6) and 94% (16/17) of patients with moderate and mild genotypes, respectively, responded to UDCA (positive or partial response) (Table S2).

### Outcome according to UDCA response

**Fig. 3:**
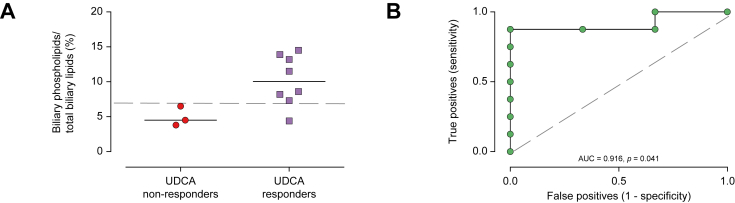
Biliary PL/TBL ratio values (median, range) in 11 patients, responders or non-responders to UDCA treatment. (A) The solid horizontal bars indicate median values. The optimal cut-off value of PL/TBL ratio predicting response to UDCA was over 6.9% (dotted horizontal line); B) ROC analysis for PL/TBL ratio values and UDCA response (sensitivity = 0.875, specificity = 1). PL, phospholipid; TBL, total biliary lipid; UDCA, ursodeoxycholic acid.

Cirrhosis was present in 48% (10/21) of patients who responded to UDCA therapy (positive or partial response) at a median age of 6.4 years (range 1.6-51.4) compared to 100% (10/10) of patients with a negative response to UDCA who exhibited cirrhosis at a median age of 6.7 years (range 3.9-9.7) (Fig. 2C; *p* = 0.0015; Fig. S1C). UDCA was started in 23 patients without cirrhosis. Progression to cirrhosis occurred in 100% (8/8), 25% (1/4) and 27% (3/11) of those with a negative, a partial and a positive response to UDCA, respectively. All patients (10/10) with a negative response to UDCA underwent LT at a median age of 7.7 years (range 4.3–10.3) compared to 5% (1/21) of the patients who responded to UDCA (Fig. 2D; *p* <0.0001; Fig. S1D). This single patient (patient 15) had a partial response to UDCA and underwent LT at the age of 30.8 years after developing a hepatocellular carcinoma, 20 years after beginning treatment. Cirrhosis-free and native liver survivals were not significantly different between patients with partial and positive response to UDCA (*p* = 0.0559 and *p* = 0.3173, respectively). Interestingly, 100% (7/7) of the patients who did not receive UDCA presented with cirrhosis at a median age of 8.3 years (range 2.3-12.2) and underwent LT at a median age of 8.7 years (range 2.3-13.1). Response to UDCA (positive or partial response) was observed in 2/8 patients with negative canalicular MDR3 staining compared to 11/12 of the patients with decreased or normal canalicular MDR3 staining (Table S2). Patients who responded to UDCA (positive or partial response) had a higher biliary PL/TBL ratio (median 10.1%, range 4.4-14.5) than those who did not respond to UDCA (4.5%, range 3.8-6.5) (Table S5). In the ROC analysis, PL/TBL ratio values could discriminate patients who responded to UDCA from those who did not. The optimal cut-off value of PL/TBL ratio predicting response to UDCA was over 6.9% (AUC = 0.916, *p* = 0.041; sensitivity = 0.875, specificity = 1) (Fig. 3).

## Discussion

In this cohort of 38 patients with PFIC3, 27 patients (71%) developed cirrhosis which manifested mainly as PHT, including 17 (45%) who were transplanted before the age of 13.5 years, confirming the severity of this disease.^1^^,^^3^^,^[7], [8], [9], [10], [11], [12] As previously reported,^11^ prolonged jaundice observed in 17 patients represented a pejorative factor since all of them underwent LT. Within this cohort, the range of PFIC3 severities was also illustrated by age at first symptoms, which ranged from 0.1 to 23.3 years: in 18 patients first symptoms were observed after age 1, including seven in whom first symptoms were observed after age 10. The outcome of these patients ranged from LT at age 2.3 years to survival with native liver up to the age of 53.8 years. The diversity of the manifestation related to MDR3 deficiency was also observed in this study, with 13 patients (34%) exhibiting evidence of gallstone disease, three of whom underwent a cholecystectomy, including two female patients experiencing nine episodes of ICP, one of whom also presented with OCIC. This diversity was also observed among the numerous relatives of the 38 patients in whom MDR3 deficiency-compatible medical events were recorded. Liver cancer and cryptogenic cirrhosis of adulthood, recorded among these relatives, three each, are now considered as belonging to the spectrum of MDR3 deficiency-related disorders.^5^^,^^6^ Liver cancer was recorded in a single patient in this cohort, but one should note that all transplanted patients underwent LT before age 13.5 years, except the patient who developed a hepatocellular carcinoma, and that the median age at last follow-up of non-transplanted patients was 12.5 years (range 3.8–53.8).

One important finding of this study is that the severity of the genotype impacts the phenotype and the outcome of patients with PFIC3. Patients with severe genotypes consistently presented with negative canalicular MDR3 staining, a negative response to UDCA therapy, and all underwent LT. Compared to patients with severe genotypes, patients with mild and moderate genotypes had a later onset of the disease, a higher biliary PL/TBL ratio, were more likely to respond to UDCA, and had prolonged NLS. In addition, patients with severe genotypes presented cirrhosis and underwent LT at a younger age than patients with mild or moderate genotypes. By contrast, patients carrying at least one missense variation had rather similar phenotypes and outcomes except for a trend towards a higher risk of presenting a negative response to UDCA and undergoing LT for patients with a moderate rather than mild genotype.

Beside the relatively low number of patients with moderate genotypes, which may have limited the power of the study, this absence of a clear-cut difference among patients with moderate and mild genotypes might be explained by the fact that missense variations are responsible for a wide range of alterations of MDR3 expression and PL transport at the canalicular level of hepatocytes.[17], [18], [19] Based on *in vitro* studies, a classification of MDR3 variants causing PFIC3 has been proposed, including missense variants that primarily affect the maturation, the activity, or the stability of the protein.^18^ The use of such a classification could help to further establish genotype-phenotype correlations and to select patients who could benefit from targeted pharmacotherapy in addition to UDCA therapy.^18^

Another important finding of this study, in line with previous studies,^3^^,^[8], [9], [10], [11], [12]^,^^17^ is that response to UDCA is critical for the outcome of patients with PFIC3. All patients who were either not treated or had a negative response to UDCA underwent LT. Among patients who responded to UDCA (positive or partial response), only one with a partial response underwent LT at the age of 30.8 years because of the development of a hepatocellular carcinoma. Only four patients developed cirrhosis while being treated and showing a positive response to UDCA, including two patients in whom signs of PHT tended to decrease over time and one additional patient who was poorly compliant to treatment. In one patient with a cirrhotic status before beginning UDCA, a decrease in signs of PHT and of liver fibrosis was observed during treatment (positive response). In addition, four patients without cirrhosis who responded to UDCA presented with splenomegaly at referral which regressed during treatment. The specific benefit of UDCA in MDR3 deficiency-related disorders relies upon the modification of the BA pool, enriched in UDCA, resulting in a less “toxic” (detergent) bile and in a less lithogenic bile by increasing the micellar solubilization of cholesterol.^2^^,^^3^ Also, it has been suggested that UDCA may act as a chaperone drug in patients with PFIC2 carrying missense BSEP variations retained in the endoplasmic reticulum.^20^^,^^21^ While this property of UDCA has not been studied *in vitro* on similar MDR3 missense variations, one cannot exclude that a chaperone effect might also contribute to the benefit of UDCA therapy in some patients with PFIC3. Episodes of poor compliance to UDCA therapy were recorded in seven patients during follow-up, indicating that physicians should regularly check for compliance, especially if serum liver tests worsen. Given the high risk of ICP in female patients with PFIC3 observed in this study and the accumulation of data supporting the safety of UDCA during pregnancy, UDCA therapy should be maintained in these patients during pregnancy.^22^

The major limitation of our study is its retrospective design. Hence, clinical investigations (*e.g*., histopathologic assessment) were not systematically performed at regular intervals and some parameters were not available at all time points. Therefore, time to cirrhosis may have been overestimated if liver deterioration occurred just after the prior liver assessment and before the next liver assessment. Another important limitation is that the study spans over 35 years. During this time, medical management of patients has changed, notably the oldest patients did not receive UDCA therapy. However, the relatively long follow-up time allowed us to take advantage of survival methods to analyze time to event data and overcome some of the limitations of the study’s retrospective design, including, but not limited to, some participants being event-free at the end of the follow-up observation period. By only including well-characterized patients managed in a single tertiary care center, we are confident that our data reflects the natural history of PFIC3 and UDCA’s effect in patients with PFIC3.

Given the emergence of next-generation sequencing in the diagnosis of genetic cholestasis including PFIC3, it is likely that patients with PFIC3 will be diagnosed at a younger age in the future, allowing for earlier medical treatment. Besides UDCA, treatments may include targeted pharmacotherapy and/or inhibition of the apical sodium-dependent bile acid transporter based on the off-label use of FDA- or EMA-approved drugs such as sodium and glycerol phenylbutyrate, ivacaftor in monotherapy or in combination with apical sodium-dependent bile acid transporter inhibitors.^17^^,^^19^^,^^20^^,^^23^^,^^24^ There is no ongoing trial assessing the safety and efficacy of a gene therapy strategy in patients with PFIC3. However, several preclinical studies have provided the proof of concept of the feasibility of this strategy in animal models of PFIC3.[25], [26], [27], [28] This strategy bears the highest hope in treating patients with PFIC3, especially those with a severe genotype and/or a negative response to UDCA for whom the only currently available treatment is LT. Our data showed that genotype and response to UDCA therapy predicted NLS in patients with PFIC3. Patients carrying at least one missense variation, with decreased or normal canalicular MDR3 staining, and a biliary PL/TBL ratio over 6.9% were more likely to respond to UDCA therapy and to exhibit prolonged NLS.

## Financial support

This study was funded by Vivet Therapeutics, Paris, France.

## Authors’ contributions

All authors have contributed to the Research design, interpretation of data and/or the drafting of the paper. All authors have approved the submitted version of the manuscript.

## Data availability statement

The datasets used and/or analysed during the current study are available from the corresponding author on reasonable request.

## Conflict of interest

EJ is consultant for Vivet Therapeutics and Laboratoire CTRS. EG is consultant for Vivet Therapeutics, Laboratoire CTRS, Albireo, Mirum. BB, SV and JPC are employees of Vivet Therapeutics.

Please refer to the accompanying ICMJE disclosure forms for further details.
