# Supplementary material for: Outcomes of 38 patients with PFIC3: Impact of genotype and of response to ursodeoxycholic acid therapy
Source: JHEP Rep. 2023 Jul 13;5(10):100844. doi: 10.1016/j.jhepr.2023.100844 (PMC10494458; doi:10.1016/j.jhepr.2023.100844)
Supplement: Multimedia component 1 [file mmc1.pdf]

Outcomes of 38 patients with PFIC3: Impact of genotype and of response to ursodeoxycholic acid therapy

Emmanuel Gonzales, Antoine Gardin, Marion Almes, Amaria Darmellah-Remil, Hanh Seguin, Charlotte Mussini, Stéphanie Franchi-Abella, Mathieu Duché, Oanez Ackermann, Alice Thébaut, Dalila Habes, Bogdan Hermeziu, Martine Lapalus, Thomas Falguières, Jean-Philippe Combal, Bernard Benichou, Sonia Valero, Anne Davit-Spraul, Emmanuel Jacquemin

Table of contents

Fig. S1 4

Table S1 6

Table S2 9

Table S3 19

Table S4 22

Table S5 25

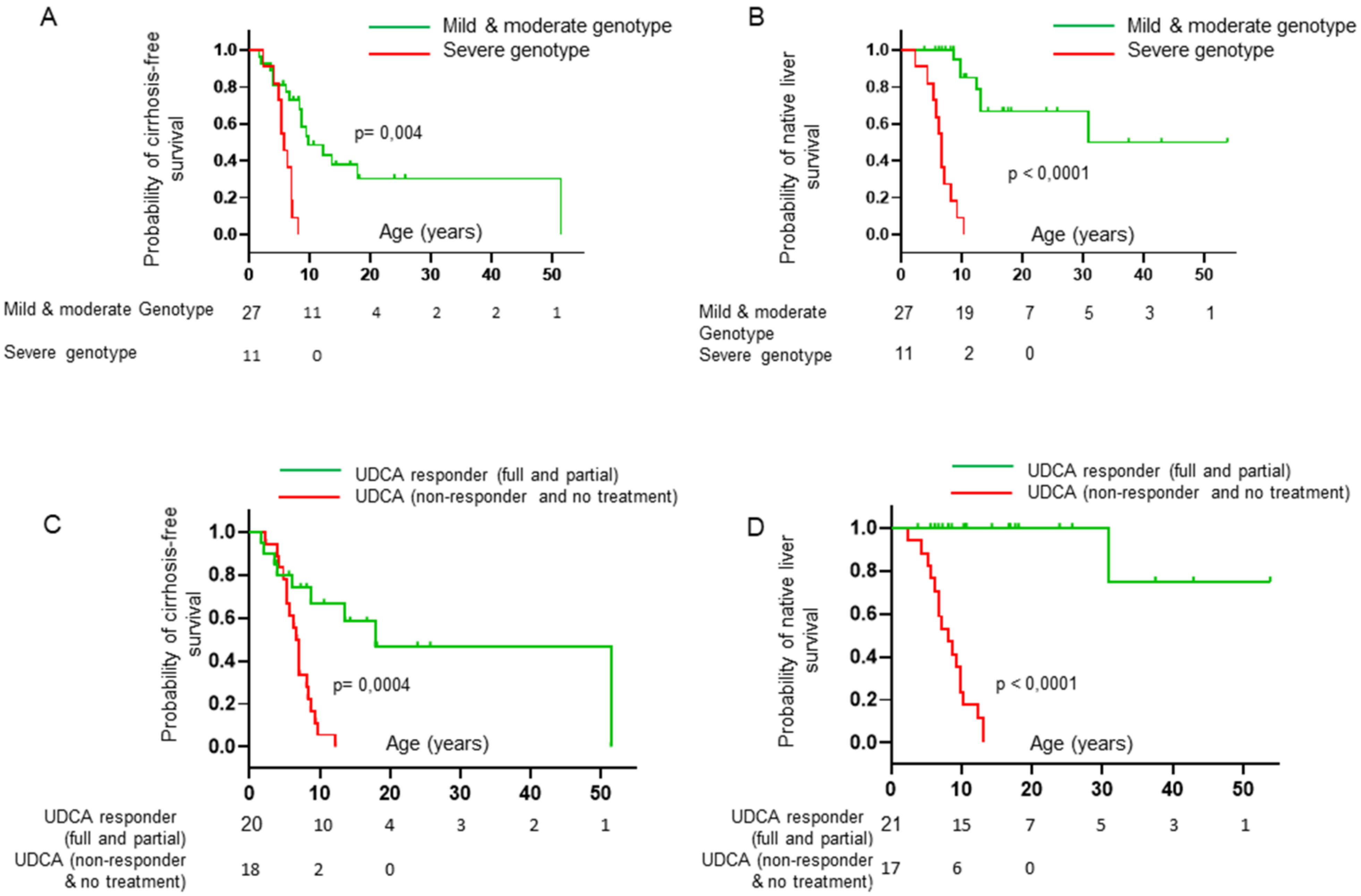
A
Probability of cirrhosis-free survival
Mild & moderate genotype
Severe genotype
p= 0,004
Age (years)
1.0
0.8
0.6
0.4
0.2
0.0
0 10 20 30 40 50
Mild & moderate Genotype 27 11 4 2 2 1
Severe genotype 11 0
B
Probability of native liver survival
Mild & moderate genotype
Severe genotype
p < 0,0001
Age (years)
1.0
0.8
0.6
0.4
0.2
0.0
0 10 20 30 40 50
Mild & moderate Genotype 27 19 7 5 3 1
Severe genotype 11 2 0
C
Probability of cirrhosis-free survival
UDCA responder (full and partial)
UDCA (non-responder and no treatment)
p= 0,0004
Age (years)
1.0
0.8
0.6
0.4
0.2
0.0
0 10 20 30 40 50
UDCA responder (full and partial) 20 10 4 3 2 1
UDCA (non-responder & no treatment) 18 2 0
D
Probability of native liver survival
UDCA responder (full and partial)
UDCA (non-responder and no treatment)
p < 0,0001
Age (years)
1.0
0.8
0.6
0.4
0.2
0.0
0 10 20 30 40 50
UDCA responder (full and partial) 21 15 7 5 3 1
UDCA (non-responder & no treatment) 17 6 0

Fig. S1: Cirrhosis-free survival and native liver survival according to genotypes and to UDCA therapy. Cirrhosis-free survival (A) (Hazard Ratio = 3.6 ; 95% CI : 1.29 – 10.05; p = 0.004, log-rank test) and native liver survival (B) (Hazard Ratio = 10.7 ; (95% CI : 2.68 – 42.79; p < 0.0001, log-rank test) per genotypic severity (patients with moderate and mild genotypes were pooled); cirrhosis-free survival (C) (Hazard Ratio = 3.5 ; 95% CI : 1.59 – 7.66 ; p = 0.0004, log-rank test) and native liver survival (D) (Hazard Ratio = 36.3 ; 95% CI : 13.48 – 97.92 ; p < 0.0001, log-rank test) per response to UDCA treatment. Patients who did not receive UDCA were pooled with non-responders. Abbreviations: UDCA, ursodeoxycholic acid.

Table S1. ABCB4 genotype and genotype classification of the 38 PFIC3 patients.

Family/ Patient	Gender	Nucleotide variation ; Amino acid change	Classification of variation	Genotype classification
A/1	F	Allele 1: c.2202dupG ; p.(Ile735AspfsX10)	Class 5	Severe
		Allele 2: c.2202dupG ; p.(Ile735AspfsX10)	Class 5	
A/2	M	Allele 1: c.2202dupG ; p.(Ile735AspfsX10)	Class 5	Severe
		Allele 2: c.2202dupG ; p.(Ile735AspfsX10)	Class 5	
A/3	F	Allele 1: c.2202dupG ; p.(Ile735AspfsX10)	Class 5	Severe
		Allele 2: c.2202dupG ; p.(Ile735AspfsX10)	Class 5	
B/4	F	Allele 1: c.1712delT ; p.(Val571AspfsX16)	Class 5	Severe
		Allele 2: c.1712delT ; p.(Val571AspfsX16)	Class 5	
C/5	M	Allele 1: c.1906 C>T ; p.(Gln636X)	Class 5	Severe
		Allele 2: c.1906 C>T ; p.(Gln636X)	Class 5	
C/6	F	Allele 1: c.1906 C>T ; p.(Gln636X)	Class 5	Severe
		Allele 2: c.1906 C>T ; p.(Gln636X)	Class 5	
D/7	M	Allele 1: c.2869C>T ; p.(Arg957X)	Class 5	Severe
		Allele 2: c.2869C>T ; p.(Arg957X)	Class 5	
E/8	M	Allele 1: c.79A>G ; p.(?)	Class 5[¤]	Severe
		Allele 2: c.79A>G ; p.(?)	Class 5[¤]	
F/9	F	Allele 1: c.1712delT ; p.(Val571AspfsX16)	Class 5	Severe
		Allele 2: c.1712delT ; p.(Val571AspfsX16)	Class 5	

**Family/ Patient**	**Gender**	**Nucleotide variation ; Amino acid change**	**Classification of variation**	**Genotype classification**
G/10	M	Allele 1: c.1712delT ; p.(Val571AspfsX16)	Class 5	Severe
		Allele 2: c.139C>T ; p.(Arg47X)	Class 5	
H/11	M	Allele 1: c.3081+5G>C ; p.(?)	Class 4	Severe
		Allele 2: c.3081+5G>C ; p.(?)	Class 4	
I/12	M	Allele 1: c.2132T>C ; p.(Phe711Ser)	Class 5	Moderate
		Allele 2: c.1006-28_1435del ; (p.?)	Class 5	
J/13	F	Allele 1: c.1270A>G ; p.(Thr424Ala)	Class 4	Moderate
		Allele 2: c.2925-10_2925-9insC ; p.(?)	Class 4	
K/14	F	Allele 1: c.344+2T>C ; p.(?)	Class 5	Moderate
		Allele 2: c.2800G>A ; p.(Ala934Thr)	Class 4	
L/15	M	Allele 1: c.2943-2952del ; p.(Phe982TrpfsX3)	Class 5	Moderate
		Allele 2: c.2947 G>A ; p.(Gly983Ser)	Class 5	
M/16	M	Allele 1: c.412T>C ; p.(Trp138Arg)	Class 5	Moderate
		Allele 2: c.3486+1063 ins148bp ; p.(?) p.Lys1163LeufsX4	Class 4	
N/17	M	Allele 1: c.2T>C ; p.(?)	Class 5	Moderate
		Allele 2: c.2876G>T ; p.(Gly959Val)	Class 5	
O/18	F	Allele 1: c.2800G>A ; p.(Ala934Thr)	Class 4	Moderate
		Allele 2: c.2924+1G>A ; p.(?)	Class 5	
P/19	F	Allele 1: c.2800G>A ; p.(Ala934Thr)	Class 4	Mild

Family/ Patient	Gender	Nucleotide variation ; Amino acid change	Classification of variation	Genotype classification
		Allele 2: c.2800G>A ; p.(Ala934Thr)	Class 4	
Q/20	M	Allele 1: c.2860G>A ; p.(Gly954Ser)	Class 4	Mild
		Allele 2: c.2860G>A ; p.(Gly954Ser)	Class 4	
R/21	M	Allele 1: c.1184A>G ; p.(Glu395Gly)	Class 4	Mild
		Allele 2: c.1184A>G ; p.(Glu395Gly)	Class 4	
S/22	M	Allele 1: c.1667T>G ; p.(Leu556Arg)	Class 4	Mild
		Allele 2: c.1667T>G ; p.(Leu556Arg)	Class 4	
T/23	F	Allele 1: c.2564A>T ; p.(Gln855Leu)	Class 4	Mild
		Allele 2: c.2564A>T ; p.(Gln855Leu)	Class 4	
U/24	M	Allele 1: c.140G>A ; p.(Arg47Gln)	Class 4	Mild
		Allele 2: c.2317 G>C ; p.(Gly773Arg)	Class 5	
V/25	F	Allele 1: c.1072G>C ; p.(Ala358Pro)	Class 4	Mild
		Allele 2: c.1691A>G ; p.(Asp564Gly)	Class 4	
W/26	M	Allele 1: c.2860G>A ; p.(Gly954Ser)	Class 4	Mild
		Allele 2: c.2860G>A ; p.(Gly954Ser)	Class 4	
X/27	M	Allele 1: c.1373A>C ; p.(Gln458Pro)	Class 4	Mild
		Allele 2: c.2800G>A ; p.(Ala934Thr)	Class 4	
Y/28	F	Allele 1: c.1880T>C ; p.(Leu627Pro)	Class 4	Mild
		Allele 2: c.1880T>C ; p.(Leu627Pro)	Class 4	
Y/29	F	Allele 1: c.1880T>C ; p.(Leu627Pro)	Class 4	Mild

**Family/ Patient**	**Gender**	**Nucleotide variation ; Amino acid change**	**Classification of variation**	**Genotype classification**
		Allele 2: c.1880T>C ; p.(Leu627Pro)	Class 4	
Z/30	M	Allele 1: c.3226A>T ; p.(Ser1076Cys)	Class 4	Mild
		Allele 2: c.3226A>T ; p.(Ser1076Cys)	Class 4	
AA/31	F	Allele 1: c.2800G>A ; p.(Ala934Thr)	Class 4	Mild
		Allele 2: c.1348_1353del ; p.(Glu450_Gly451del)	Class 4[#]	
AB/32	M	Allele 1: c.2800G>A ; p.(Ala934Thr)	Class 4	Mild
		Allele 2: c.2932T>C ; p.(Ser978Pro)	Class 4	
AC/33	M	Allele 1: c.524C>A ; p.(Thr175Lys)	Class 4	Mild
		Allele 2: c.524C>A ; p.(Thr175Lys)	Class 4	
AD/34	M	Allele 1: c.431G>A ; p.(Arg144Gln)	Class 4	Mild
		Allele 2: c.3233T>A ; p.(Val1078Glu)	Class 4	
AE/35	M	Allele 1: c.1295 G>A ; p.(Gly432Asp)	Class 4	Mild
		Allele 2: c.959 C>T ; p.(Ser320Phe)	Class5	
AF/36	F	Allele 1: c.140G>A ; p.(Arg47Gln)	Class 4	Mild
		Allele 2: c.1296_1301del ; p.(Cys433_Gly434del)	Class 4[#]	
AG/37	F	Allele 1: c.1529A>C ; p.(Asn510Thr)	Class 4	Mild
		Allele 2: c.1529A>C ; p.(Asn510Thr)	Class 4	
AG/38	F	Allele 1: c.1529A>C ; p.(Asn510Thr)	Class 4	Mild
		Allele 2: c.1529A>C ; p.(Asn510Thr)	Class 4	

Nucleotide variations are classified according to the American College of Medical Genetics (ACMG) guidelines. [#], In-frame deletion; [¤], Splice effect predicted in silico.

Table S2: Detailed characteristics including MDR3 canalicular staining, biliary phospholipids analysis, clinical symptoms, UDCA treatment and treatment response, cirrhotic status, and outcome of the 38 PFIC3 patients, according to genotype classification.

Patient	MDR3 canalicular staining	Biliary phospholipids (% of total biliary lipids, N 19-24%)	Age at first symptoms/at evaluation in referral center (years)	Symptoms at first evaluation in referral center	Response to UDCA/ age at UDCA initiation (years)	Evolution	Age at cirrhosis (years)	Age at 1st LT (years)	Indication for LT	Age at last f/u (years)	Status at last f/u
**Patients with severe genotype**											
A1	NA	NA	5.3/5.9	J, H, S, As	NA/NA	P, HE, liver failure<br><br>UGIE N (5.3y)<br><br>EHL	5.3 (F4, PHT)	6.7	Decompensated cirrhosis	39.6	Dead[a]
A2	NA	NA	0.8/3.3	J, H, S, P	NA/NA	$P^{(P+C)+}$, As<br><br>OVI RS- (3.9 y)	5.3 (PHT)	5.3	Decompensated cirrhosis	33.7	Alive
A3	NA	NA	0.2/0.2	H, S	Negative/ 4.9	P^{U-}, As, J<br><br>OVI RS- (4.9 y)<br><br>EHL	5.7 (PHT)	5.7	Cirrhosis and pruritus<br>re-LT: cirrhosis due to biliary strictures<br><br>Pregnancy x1, live birth x1	30.4	Alive
B4	Absent	NA	1.1/4.8	J, SD, H, S, P	Negative/ 4.9	$P^{U+/-, (U+C)+/-}$<br><br>OVIII (4.8y)<br>PX1<br>sclerotherapy x 1	4.8 (F4, PHT)	6.2	Decompensated cirrhosis	35.0	Alive

Patient	MDR3 canalicular staining	Biliary phospholipids (% of total biliary lipids, N 19-24%)	Age at first symptoms/at evaluation in referral center (years)	Symptoms at first evaluation in referral center	Response to UDCA/ age at UDCA initiation (years)	Evolution	Age at cirrhosis (years)	Age at 1st LT (years)	Indication for LT	Age at last f/u (years)	Status at last f/u
C5	Absent	2.7	0.2/1.3	J, SD, H, S, As	NA/NA	HPS (1.3 y)<br><br>OVI RS- (1.3 y)	2.3 (F4, PHT)	2.3	Decompensated cirrhosis, HPS	28.0	Alive
C6	Absent	NA	0.1/0.1	J, SD, H, S, P	Negative/ 0.3	$P^{U+/-, (U+R)+}$, As<br><br>OVI RS- (0.3 y, 0.9 y, 1.9 y)	4.1 (F4, PHT)	4.3	Decompensated cirrhosis<br>re-LT: chronic rejection	29.1	Alive
D7	Absent	NA	0.7/2.8	Transient neonatal J, H, S, P	Negative/ 2.8	$P^{(U+R)+/-}$, J, liver failure<br><br>OVII RS- (9 y)	8.1 (PHT)	9.2	Decompensated cirrhosis<br>Re-LT: HAT and graft dysfunction	24.1	Dead[b]
E8	Absent	NA	0.2/3.2	Transient neonatal J, H, S, P	Negative/ 7.1	P^{U+}, J, As, liver failure<br><br>OVII RS+ (9.2 y), PX1 ligation x 3	7.1 (F4, PHT)	10.3	Decompensated cirrhosis	18.0	Alive
F9	NA	4.5	0.8/0.8	Transient neonatal J, P	Negative[C] /0.8	P^{U+}, J, H, S, HE, As, liver failure<br><br>OVI (7y)	7.0 (PHT)	7.1	Decompensated cirrhosis<br>re-LT: HAT, cholangiopathy	20.8	Alive
G10	Absent	3.8	5.7/5.7	H, S, P	Negative/ 5.7	$P^{(U+R)+}$, J, As, liver failure<br><br>OVII (7.8y), PX1 1igation x 1	7.0 (PHT)	8.2	Decompensated cirrhosis	9.2	Alive
H11	NA	NA	0.3/1.2	Transient neonatal J, P, H	Negative/ 1.2	$P^{U-, (U+R)+/-}$, J, S,<br><br>UGIE N (4.5y)	6.3 (F4, PHT)	6.7	Cirrhosis and pruritus	7.8	Alive

Patient	MDR3 canalicular staining	Biliary phospholipids (% of total biliary lipids, N 19-24%)	Age at first symptoms/at evaluation in referral center (years)	Symptoms at first evaluation in referral center	Response to UDCA/ age at UDCA initiation (years)	Evolution	Age at cirrhosis (years)	Age at 1st LT (years)	Indication for LT	Age at last f/u (years)	Status at last f/u
**Patients with moderate genotype**											
I12	NA	NA	13.6/13.7	H, S, P, As, Bl	Positive[T]/ 13.9	P[U+], S, Bl<br><br>OVII RS+ (13.7y), PX2 sclerotherapy x12 - UGIE OVI (27 y)	13.6 (F4, PHT)	No LT		42.9	Alive
J13	Decreased	4.7	0.8/12.6	H, S, P, J, Bl, OVII RS+ (9.4 y) -SPSD at 9.4y)	NA/ NA	P[C+], liver failure	9.4 (F4, PHT)	13.1	Decompensated cirrhosis	39.7	Alive
K14	Decreased	7.3	17.6/17.9	J, P, OCIC, EHL	Positive[C]/ 17.9	P[U+/-], regression of jaundice, S<br><br>UGIE N (17.9 y)<br><br>EHL, cholecystectomy at 17.9 y<br><br>Pregnancy x 3 ICP x3 live birth x 3	17.9 (F4)	No LT		37.5	Alive
L15	NA	14.5	0.7/0.7	H, S, P, J	Partial/ 10.4	P[C+/-, (C+U)+], intermittent J, transient As	6.7 (PHT)	30.8	Cirrhosis and HCC	31.4	Alive

Patient	MDR3 canalicular staining	Biliary phospholipids (% of total biliary lipids, N 19-24%)	Age at first symptoms/at evaluation in referral center (years)	Symptoms at first evaluation in referral center	Response to UDCA/ age at UDCA initiation (years)	Evolution	Age at cirrhosis (years)	Age at 1st LT (years)	Indication for LT	Age at last f/u (years)	Status at last f/u
						OVI (6.7 y) PX1 – OGV2 (27.7 y)					
M16	Absent	6.5	2/3.9	SD, H, S, P	Negative/ 4.5	P^{U+}, As, J, liver failure<br><br>OVII RS- (9.8 y)	3.9 (F4, PHT)	9.8	Decompensated cirrhosis<br>Re-LT: HAT, cholangiopathy	27.6	Alive
N17	NA	NA	15.7/16.4	EHL cholecystectomy at 15.7y	Partial^{C}/ 16.4	no symptom<br><br>no endoscopy	no cirrhosis	No LT		18.1	Alive
O18	Decreased	13.9	3.9/3.9	H, S, P	Positive/ 4.2	P^{U+}<br><br>OVIII RS+ (4.1 y), PX1 ligation x 2 - UGIE N (9.4 y), liver/spleen stiffness 15.4/26.5 kPa at 10.3y	3.9 (F4, PHT)	No LT		10.3	Alive
**Patients with mild genotype**											
P19	Normal	NA	23.3/26.3	J, P, EHL/LPAC, Cholecystectomy at 25.9 y, ICP	Partial^{C}/ 27.3	P^{U+}, transient SD<br><br>UGIE N (45.4 y)<br><br>LPAC (multiple endoscopic	51.4<br><br>(PHT, dysmorphic liver at imaging)	No LT		53.8	Alive

Patient	MDR3 canalicular staining	Biliary phospholipids (% of total biliary lipids, N 19-24%)	Age at first symptoms/at evaluation in referral center (years)	Symptoms at first evaluation in referral center	Response to UDCA/ age at UDCA initiation (years)	Evolution	Age at cirrhosis (years)	Age at 1st LT (years)	Indication for LT	Age at last f/u (years)	Status at last f/u
						retrograde procedures)<br><br>Pregnancy x 7, ICP x6, live birth x5,					
Q20	NA	NA	4.0/12.7	J, H, S, P, As, VOI	NA/NA	P^{C-}, liver failure<br><br>OVI (12.8 y)	12.2 (F4, PHT)	13.1	Decompensated cirrhosis<br>Re-LT: Unknown	39.5	Dead[c]
R21	NA	NA	5.1/9.8	Transient J, H, S, Bl at 5.1y, As, EHL	NA/NA	Bl, J, liver failure<br><br>OVIII RS+ (8.9 y), PX2 sclerotherapy x 1- SPSD at 11.7y<br><br>EHL	8.7 (F4, PHT)	12.4	Decompensated cirrhosis	36.9	Alive
S22	NA	NA	0.4/6.9	SD, H, S, P	NA/ NA	$P^{C+/-}$, J, As, liver failure<br><br>OVI (8.3 y)	8.3 (PHT)	8.7	Decompensated cirrhosis	35.6	Alive
T23	Decreased	8.2	1.7/2.6	H, P	Partial[T] /2.6	P^{U+}, transient J, transient SD,<br><br>UGIE N (2.6 y)<br><br>LPAC/EHL	no cirrhosis	No LT		25.7	Alive

Patient	MDR3 canalicular staining	Biliary phospholipids (% of total biliary lipids, N 19-24%)	Age at first symptoms/at evaluation in referral center (years)	Symptoms at first evaluation in referral center	Response to UDCA/ age at UDCA initiation (years)	Evolution	Age at cirrhosis (years)	Age at 1st LT (years)	Indication for LT	Age at last f/u (years)	Status at last f/u
U24	Normal	NA	10.4/10.8	P	Positive^{C}/ 11.9	P$^{U+,}$ no symptom<br><br>UGIE N (11.8y)<br><br>LPAC	no cirrhosis	No LT		23.9	Alive
V25	NA	NA	0.8/3.2	H, S, P	Positive^{T}/ 5.2	P^{U+}, H, regression of S<br><br>UGIE OVI (6.1y), (PX1) - N (11.2y)	6.1 (PHT)	No LT		17.6	Alive
W26	Decreased	NA	0.5/2.8	H, S, P	Positive^{T}/ 2.9	P^{U+}, regression of H and S, no symptom<br><br>no endoscopy	1.6 (PHT)	No LT		16.9	Alive
X27	Decreased	8.6	7.2/9	None (isolated abnormal liver tests)	Positive^{C}/ 9.1	no symptoms<br><br>no endoscopy, liver stiffness 6,9 kPa at 13 y<br><br>Transient IHL/LPAC	no cirrhosis	No LT		16.7	Alive
Y28	Decreased	NA	0.7/2.5	H, S, P	Positive/ 2.6	P^{U+}, regression of H and S, no symptom	no cirrhosis	No LT		14.3	Alive

Patient	MDR3 canalicular staining	Biliary phospholipids (% of total biliary lipids, N 19-24%)	Age at first symptoms/at evaluation in referral center (years)	Symptoms at first evaluation in referral center	Response to UDCA/ age at UDCA initiation (years)	Evolution	Age at cirrhosis (years)	Age at 1st LT (years)	Indication for LT	Age at last f/u (years)	Status at last f/u
						no endoscopy, liver stiffness 6kPa at 14.3 y					
Y29	NA	NA	1.3/1.8	H, S, P	Partial/ 2.3	P[U+], H, regression of S<br><br>no endoscopy, liver stiffness 5 kPa at 5.6y	no cirrhosis	No LT		5.6	Alive
Z30	Normal	NA	0.6/1.3	P, J, SD, H	Negative[C] / 1.4	P[(U+R)+], liver failure<br><br>UGIE N (5y)	9.7 (F4)	9.8	Decompensated cirrhosis	12.7	Alive
AA31	Absent	NA	6.8/8.5	None (isolated abnormal liver tests)	Positive/ 8.9	no symptom<br><br>no endoscopy, liver/spleen stiffness 5.9/15 kPa at 10.5 y<br><br>Transient EHL	no cirrhosis	No LT		10.6	Alive
AB32	Decreased	13.2	0.1/3.4	H, S, P	Positive/ 3.5	P[U+/-, (U+R)+/-]<br><br>UGIE N at 3.5 y and at 5.2 y, OVI (8.7y), liver/spleen stiffness 13.4/25.9 kPa at 8.6 y	8.7 (PHT)	No LT		10.7	Alive

Patient	MDR3 canalicular staining	Biliary phospholipids (% of total biliary lipids, N 19-24%)	Age at first symptoms/at evaluation in referral center (years)	Symptoms at first evaluation in referral center	Response to UDCA/ age at UDCA initiation (years)	Evolution	Age at cirrhosis (years)	Age at 1st LT (years)	Indication for LT	Age at last f/u (years)	Status at last f/u
AC33	Normal	4.4	0.9/1.9	H, P, S	Positive/ 2.2	P^{U+}<br><br>OVII, RS+ (4.2 y) - PX1 ligation x 3, UGIE severe hypertensive gastropathy no OV at 7.6y, liver/spleen stiffness 6.9/41 kPa at 8.1	2.0 (F4, PHT)	No LT		8.6	Alive
AD34	Decreased	NA	0.5/2	H, S, P, EHL	Positive/ 2.1	P^{U+}, regression of S<br><br>UGIE N (2.1 y), liver/spleen stiffness 8.7/24.1 kPa at 7.2 y<br><br>EHL	no cirrhosis	No LT		7.3	Alive
AE35	Normal	11.5	0.5/1	H, P, S	Positive/ 1.2	P^{U+}, regression of S<br><br>UGIE N (1.1 y), liver/spleen stiffness 9.6/21.5 kPa at 6.5 y	3.5 (PHT)	No LT		6.7	Alive
AF36	NA	NA	0.9/1	H	Positive/ 1.3	no endoscopy, liver/spleen stiffness	no cirrhosis	No LT		6.2	Alive

Patient	MDR3 canalicular staining	Biliary phospholipids (% of total biliary lipids, N 19-24%)	Age at first symptoms/at evaluation in referral center (years)	Symptoms at first evaluation in referral center	Response to UDCA/ age at UDCA initiation (years)	Evolution	Age at cirrhosis (years)	Age at 1st LT (years)	Indication for LT	Age at last f/u (years)	Status at last f/u
						7.9/22.5 kPa at 6.2 y					
AG37	Absent	NA	4.7/5.3	H, S, P	Positive/ 5.5	P^{U+}, regression of S<br><br>UGIE N 5.7 y, liver/spleen stiffness 6.9/19.4 kPa at 8.1 y	no cirrhosis	No LT		8.1	Alive
AG38	NA	NA	1.8/1.8	H, P (familial screening)	Positive/ 1.9	P^{U+}, regression of H<br><br>no endoscopy, liver/spleen stiffness 6/23.3 kPa at 3.8 y	no cirrhosis	No LT		3.8	Alive

Abbreviations: As, ascites; Bl, gastrointestinal bleeding due to portal hypertension; EHL, extrahepatic biliary lithiasis; F4, cirrhosis at the pathological examination of liver sample allowing to establish the status of cirrhosis; f/u, follow-up; H, hepatomegaly; HAT, hepatic artery thrombosis; HCC, hepatocellular carcinoma; HE, hepatic encephalopathy; HPS, hepatopulmonary syndrome; J, jaundice; LT, liver transplantation; NA, not available or not applicable; OCIC, oral contraceptive induced cholestasis; OGV-2, oesophago-gastric varice type 2; OV, oesophageal varice; P, pruritus; P^{U+}, total relief of pruritus with UDCA; P^{R+}, total relief of pruritus with rifampicin; P^{C+}, total relief of pruritus with cholestyramine; $P^{(U+R)+}$, total relief of pruritus with UDCA and rifampicin; $P^{(P+C)+}$, total relief of pruritus with phenobarbital and cholestyramine; $P^{(U+C)+}$, total relief of pruritus with UDCA and cholestyramine; $P^{U+/-}$, partial relief of pruritus with UDCA; $P^{R+/-}$, partial relief of pruritus with rifampicin; $P^{C+/-}$, partial relief of pruritus with cholestyramine; $P^{(U+R)+/-}$, partial relief of pruritus with UDCA and rifampicin; $P^{(U+C)+/-}$, partial relief of pruritus with UDCA and

cholestyramine; P^{U-}, no improvement of pruritus with UDCA; P^{R-}, no improvement of pruritus with rifampicin; P^{C-}, no improvement of pruritus with cholestyramine; PHT, portal hypertension; Re-LT, retransplantation; RS, red sign; S, splenomegaly; SD, stool discoloration; SPSD, surgical portosystemic derivation; TJ, transient jaundice; UGIE, upper gastrointestinal endoscopy; a, Squamous cell carcinoma of the oral cavity; b, Multiple organ failure following liver retransplantation; c, Colon cancer; C, doubt on compliance to UDCA; T, UDCA discontinuation test.

Table S3. Main biological parameters of the 38 PFIC3 patients at the time of referral and at last follow-up with native liver.

Patient	timing of lab test	Alb (g/L)	BiliT (µmol/L)	BiliC (µmol/L)	ALT (U/L)	GGT (U/L)	sBA (µmol/L)	aFP (ng/mL)	PLT (10e9/L)	PT (%)	Fact V (%)
1	first evaluation	38	119	71	187	318	na	na	216	37	35
1	at last follow-up	43	294	168	63	122	na	na	130	38	43
2	first evaluation	43	68	51	126	668	na	na	174	72	100
2	at last follow-up	na	421	328	324	540	na	na	58	87	na
3	first evaluation	na	54	37	105	885	na	na	549	91	100
3	at last follow-up	26	148	102	515	153	na	5	156	53	100
4	first evaluation	40	147	105	372	761	40	6	90	96	100
4	at last follow-up	37	100	78	205	271	na	na	na	100	100
5	first evaluation	35	74	48	510	522	na	10	110	na	na
5	at last follow-up	na	71	na	109	387	na	na	110	89	83
6	first evaluation	41	56	43	44	285	50	5	495	100	na
6	at last follow-up	39	194	144	351	390	na	5	130	100	100
7	first evaluation	46	33	22	137	958	na	na	na	91	100
7	at last follow-up	31	521	378	129	306	212	na	86	34	38
8	first evaluation	na	10	5	76	141	na	na	251	100	na
8	at last follow-up	34	69	56	193	68	na	na	62	33	24
9	first evaluation	na	570	na	107	na	na	na	na	17	16
9	at last follow-up	19	431	254	156	149	na	na	126	21	18
10	first evaluation	39	33	27	368	484	304	na	166	100	na
10	at last follow-up	na	170	125	104	27	495	na	52	44	29
11	first evaluation	na	21	17	429	700	na	7	434	81	na
11	at last follow-up	45	252	191	120	662	na	na	126	71	na
12	first evaluation	29	11	6	79	83	71	6	71	74	65
12	at last follow-up	37	na	12	40	25	na	6	38	69	na
13	first evaluation	27	205	105	108	323	na	na	155	61	50
13	at last follow-up	na	126	33	336	81	na	na	122	na	54
14	first evaluation	38	112	69	480	150	na	na	209	80	na
14	at last follow-up	na	10	na	N	37	na	na	124	86	na
15	first evaluation	52	70	na	214	223	na	na	na	51	na

15	at last follow-up	na	42	28	57	166	na	na	57	71	na
16	first evaluation	41	23	na	149	103	95	na	175	85	100
16	at last follow-up	32	384	238	103	123	na	na	25	31	41
17	first evaluation	47	6	na	957	395	na	na	376	100	na
17	at last follow-up	na	5	na	99	29	na	3	na	na	na
18	first evaluation	41	29	20	258	782	98	1	152	100	100
18	at last follow-up	43	13	na	15	14	41	1	105	82	80
19	first evaluation	na	21	na	55	680	na	na	na	98	na
19	at last follow-up	na	9	na	49	1379	na	na	159	100	na
20	first evaluation	24	328	245	122	116	180	5	105	50	44
20	at last follow-up	na	na	na	na	na	na	na	53	36	30
21	first evaluation	na	23	na	193	385	na	na	117	85	na
21	at last follow-up	41	120	78	33	na	na	na	62	53	42
22	first evaluation	na	26	18	na	450	na	na	134	na	na
22	at last follow-up	28	763	480	161	272	na	na	na	31	32
23	first evaluation	na	21	4	394	81	na	na	243	100	100
23	at last follow-up	52	22	5	85	49	na	na	na	100	na
24	first evaluation	38	9	na	113	78	na	na	378	100	na
24	at last follow-up	48	15	3	83	72	na	2	338	100	na
25	first evaluation	47	21	6	99	218	89	na	232	100	96
25	at last follow-up	53	20	6	18	21	11	5	165	93	80
26	first evaluation	49	7	1	268	128	15	5	470	100	100
26	at last follow-up	45	9	3	20	10	11	na	296	100	na
27	first evaluation	38	7	na	136	106	na	2	231	100	100
27	at last follow-up	43	28	9	175	370	3	1	na	89	85
28	first evaluation	na	13	na	154	105	na	3	179	81	na
28	at last follow-up	41	22	7	11	11	na	na	na	94	90
29	first evaluation	43	20	15	259	100	186	na	na	100	100
29	at last follow-up	44	13	5	54	76	15	na	248	100	100
30	first evaluation	na	64	21	460	483	215	na	475	na	na
30	at last follow-up	34	227	194	177	1271	na	6	196	69	57
31	first evaluation	na	17	6	153	165	na	na	247	89	na
31	at last follow-up	48	15	na	17	8	13	1	261	93	100
32	first evaluation	43	5	na	566	806	72	2	151	100	86

32	at last follow-up	41	6	na	24	26	13	1	121	85	81
33	first evaluation	48	6	na	460	278	na	na	155	100	90
33	at last follow-up	46	11	na	22	10	14	na	66	68	36
34	first evaluation	42	23	12	53	230	99	3	250	82	94
34	at last follow-up	49	17	na	37	17	25	3	324	84	na
35	first evaluation	39	4	2	201	75	37	4	446	84	na
35	at last follow-up	45	6	na	19	10	18	na	298	89	60
36	first evaluation	na	5	na	180	217	na	na	346	100	na
36	at last follow-up	48	4	na	18	7	18	1	372	87	na
37	first evaluation	35	17	9	150	216	na	1	na	94	na
37	at last follow-up	45	4	na	34	8	20	1	208	95	na
38	first evaluation	na	7	na	226	152	na	na	141	80	na
38	at last follow-up	37	4	1	38	14	4	2	257	96	na

Abbreviations: aFP, serum alpha foetoprotein concentration (normal value <10 ng/mL) ; Alb, serum albumin (normal value 35-52 g/L); ALT, serum alanine aminotransferase activity (normal value <50 IU/L); BiliC, serum conjugated bilirubin (normal value <5 µmol/L); BiliT, serum total bilirubin (normal value <17 µmol/L); Fact V, clotting factor V (normal value >80%); GGT, serum γ-glutamyl transferase activity (normal value <40 IU/L); sBA, serum bile acid concentration (normal value <10 µmol/L); PLT, platelet count (normal value 1.5-4 G/L); PT, prothrombin time (normal value >70%).

Table S4. Histological analysis of 58 liver samples from 34 patients with PFIC3.

	Age at assessment (years)	Metavir fibrosis score	Metavir activity score	Ductular proliferation	Ductular plug	Giant cell Transformation	Cholestasis (hepatocellular and/or canalicular)
1	5.4	F4	A1	+	-	-	+
2	1.3	F1/F2	A0/A1	+	-	-	-
2	2.4	F2	A0/A1	+	-	-	-
3	0.3	F1/F2	NA	-	-	-	+
4	2.3	F2	A1	+	-	-	+
4	5.0	F4	A1	-	-	-	-
4[£]	6.2	F4	NA	-	+	-	+
5	0.9	F1/F2	NA	-	-	-	-
5[£]	2.3	F4	A2/A3	+	+	-	+
6	0.3	F1/F2	A1	+	-	+	+
6[£,#]	4.3	F4	A2	+	+	+	+
7[*]	3.1	F3/F4	A1/A2	+	-	-	-
7[*,£]	9.2	F4	A2	+	+	-	+
8	3.2	F3	NA	+	-	-	-
8	7.1	F4	A2	+	-	-	+
8[*,£]	10.3	F4	A1	+	-	-	+
9*	1.2	F3	NA	+	-	-	-
10[*]	7.0	F3/F4	A1	+	-	-	+
10[*,£]	8.2	F4	A1	+	+	+	+
11[*]	6.5	F4	A2	+	+	-	+
11[*,£]	6.7	F4	NA	-	-	-	+

	Age at assessment (years)	Metavir fibrosis score	Metavir activity score	Ductular proliferation	Ductular plug	Giant cell Transformation	Cholestasis (hepatocellular and/or canalicular)
12	13.7	F4	A0/A1	+	-	-	-
12	13.9	F4	A0/A1	+	+	-	-
13	4.8	F1	A0/A1	+	-	-	-
13	5.1	F1/F2	A0/A1	+	-	-	-
13	5.6	F3/F4	A0/A1	+	-	-	+
13	9.4	F4	A0/A1	+	-	+	+
14[*]	17.9	F4	A1/A2	+	-	+	+
15	1.7	F1/F2	NA	+	-	-	-
15	10.4	F4	A2	+	+	-	+
16	4.5	F4	A1/A2	+	-	-	+
16[*,£]	9.8	F4	A2	+	+	-	+
18	4.2	F4	A2	+	-	-	-
19	27.2	F0	A0	-	-	-	-
19[*]	48.8	F1/F2	A1/A2	+	-	-	-
20	12.2	F4	A1/A2	+	-	-	+
20[£]	13.1	F4	A1/A2	+	+	-	+
21	9.0	F4	A2/A3	+	-	-	-
22	0.7	F1	NA	-	-	-	-
22	1.4	F1	A0/A1	-	-	-	-
22	3.2	F2	A1	-	-	-	-
22	6.9	F3/F4	A1	-	-	-	+
23	2.6	F2	A1	+	-	-	-
24	11.9	F2	A0/A1	+	-	-	-

	Age at assessment (years)	Metavir fibrosis score	Metavir activity score	Ductular proliferation	Ductular plug	Giant cell Transformation	Cholestasis (hepatocellular and/or canalicular)
25	3.2	F3/F4	A1/A2	+	-	-	-
25	3.4	F3/F4	A1	+	-	-	-
26	2.2	F3/F4	A2	+	-	-	-
26	2.9	F3/F4	A1	-	-	-	-
26*	5.0	F2/F3	A0/A1	+	-	-	-
27	9.1	F2	A0/A1	+	-	-	-
28*	3.6	F3/F4	A1/A2	-	-	-	-
30*,£,#	9.8	F4	A1/A2	+	+	-	+
31	8.9	F3	A1/A2	+	-	-	-
32	3.5	F3/F4	A2	+	+	-	-
33	2.0	F4	A1	+	-	-	+
34	2.1	F3/F4	A1/A2	+	-	-	-
35	1.2	F2/F3	A2/A3	+	-	-	-
37*	5.7	F1	A0/A1	+	-	-	-

Abbreviations: +, present; -, absent; £, whole native liver at the time of LT; *, biopsy performed while the patient was receiving UDCA; NA, data allowing the calculation of an activity score were not available in the pathology report. #, presence of intrahepatic lithiasis that was not previously identified by imaging. Lines highlight in grey indicate liver samples that were obtained before, at the time, or shortly after referral.

Table S5. Biliary lipid composition in 13 PFIC3 patients.

Patient	BA (mmol/L)	Ch (mmol/L)	PL (mmol/L)	TBL (mmol/L)	Ch/PL (N: 0.1-0.16)	BA/PL (N: 3-5)	PL/TBL (%) (N: 19-24%)
5[£,*]	3.5	0.04	0.10	3.64	0.40	35.0	2.7
9[£]	6.0	0.41	0.30	6.71	1.37	20.0	4.5
10[#]	1.9	0.33	0.09	2.34	3.67	21.3	3.8
13[£,*]	9.9	0.20	0.50	10.6	0.40	19.8	4.7
14[£]	50.6	1.70	4.10	56.4	0.41	12.3	7.3
15[£]	6.8	0.29	1.20	8.29	0.24	5.7	14.5
16[£]	7.0	1.70	0.60	9.3	2.83	11.7	6.5
18[£]	86.0	3.15	14.40	103.55	0.22	6.0	13.9
23[£]	36.5	0.90	3.00	36.64	0.30	12.2	8.2
27[£]	92.5	2.25	8.90	103.95	0.25	10.4	8.6
32[$]	30.6	1.54	4.89	37.03	0.31	6.3	13.2
33[£]	47.5	0.78	2.24	50.52	0.35	21.2	4.4
35[£]	33.0	0.68	4.36	38.04	0.16	7.6	11.5

Abbreviations: BA, biliary bile acid; Ch, biliary cholesterol; N, control value (reference 1); PL, biliary phospholipid; TBL, total biliary lipids (calculated as the sum of BA, Ch and PL). [£], gallbladder bile sample obtained during a percutaneous transhepatic cholecystography; [#], gallbladder bile sample obtained at time of the liver transplantation; [$], choledochal bile obtained during an endoscopic retrograde cholangiopancreatography; *, Patient who did not receive ursodeoxycholic acid treatment.
